# All-optical broadband ultrasonography of single cells

**DOI:** 10.1038/srep08650

**Published:** 2015-03-03

**Authors:** T. Dehoux, M. Abi Ghanem, O. F. Zouani, J.-M. Rampnoux, Y. Guillet, S. Dilhaire, M.-C. Durrieu, B. Audoin

**Affiliations:** 1 Univ. Bordeaux, I2M, UMR 5295, F-33400 Talence, France; 2 CNRS, I2M, UMR 5295, F-33400 Talence, France; 3 Univ. Bordeaux, CBMN, UMR CNRS 5248, F-33607 Pessac, France; 4 Univ. Bordeaux, LOMA, CNRS UMR 5798, F-33400 Talence, France

## Abstract

Cell mechanics play a key role in several fundamental biological processes, such as migration, proliferation, differentiation and tissue morphogenesis. In addition, many diseased conditions of the cell are correlated with altered cell mechanics, as in the case of cancer progression. For this there is much interest in methods that can map mechanical properties with a sub-cell resolution. Here, we demonstrate an inverted pulsed opto-acoustic microscope (iPOM) that operates in the 10 to 100 GHz range. These frequencies allow mapping quantitatively cell structures as thin as 10 nm and resolving the fibrillar details of cells. Using this non-invasive all-optical system, we produce high-resolution images based on mechanical properties as the contrast mechanisms, and we can observe the stiffness and adhesion of single migrating stem cells. The technique should allow transferring the diagnostic and imaging abilities of ultrasonic imaging to the single-cell scale, thus opening new avenues for cell biology and biomaterial sciences.

The stiffness and the adhesion strength of cells have been recognized as key players in many fundamental processes, such as mechano-transduction^1^, morphogenesis^2^, motility^3^,^4^ and progression of degenerative diseases^5^,^6^. Existing modalities that can map these properties with a sub-cell resolution use contact probes, including atomic force microscopy^7^ and pressurized nanopipette^8^, or nanostructured substrates as a two-dimensional array of contact probes^9^,^10^,^11^. Such analysis may interfere with normal cellular functions^12^, and requires a complex modeling of the cell-probe interaction, hampered by the contribution of the cell structure, such as the plasma membrane^13^.

As a non-invasive alternative to contact-based modalities, ultrasonic techniques offer time-resolved mapping of the mechanical properties of tissues. In classical ultrasonography, an ultrasound pulse is sent into the body. This pulse reflects off interfaces between tissues with contrasted acoustic impedances, and it carries back information on stiffness, viscosity and topography of tissues. Using piezo-electric transducers in contact with the tissue to generate and detect sound waves, typical carrying frequencies of 10 MHz are obtained, offering sub-millimetric resolution. Scanning acoustic microscopy^14^ uses an acoustic lens to focus sound down to a diffraction-limited spot. At room temperature, it has extended the frequency range up to ~ 2 GHz, offering typical resolutions of ~ 0.7 *µ*m using water as the coupling medium^15^,^16^,^17^. In cryogenic conditions, frequencies as high as 15 GHz have been reached in pressurized liquid Helium, offering a ~ 15 nm lateral resolution for metallic samples^18^. In single cells, acoustic microscopy has allowed measuring the mechanical properties at 1 GHz with a 3 *µ*m axial resolution^19^ and the evaluation of their adhesion. But even at these frequencies it is difficult to distinguish the fine structure of a cell, in particular in thin regions such as the lamella.

In 1984, a time-resolved opto-acoustic technique called picosecond ultrasonics (PU)^20^ was developed that could implement frequencies up to a few THz thanks to the thermoelastic expansion induced by the absorption of femtosecond laser pulses. Similarly to Brillouin spectroscopy, PU uses the scattering of light by acoustic waves to probe the mechanical properties of a medium. However, contrary to Brillouin spectroscopy^21^, PU uses optical sampling to achieve time-resolved measurements, thereby probing the in-depth topography of the sample^22^. Moreover, PU takes advantages of the controlled emission of acoustic waves to amplify the light scattering, hence allowing the study of smaller volumes. Bridging acoustic microscopy and PU, an acoustic lens with a high numerical aperture^23^ combined with a Fabry-Perot cavity^24^ has been recently developed to create a new type of acoustic microscope offering a 100 nm lateral resolution at room temperature.

Primarily dedicated to the investigation of thin solid films, the ability of PU to tackle important issues in single-cell biology was soon foreseen^25^, and the first applications to vegetal cells have recently been developed^26^,^27^. Based on this technology, we here present state-of-the-art ultrasonography at frequencies up to ~ 100 GHz to probe cell structures as thin as 10 nm, and resolve the fibrillar details of cells.

We used a method reminiscent of medical percussion, in which tapping the surface of the body reveals its underlying structure. A mat sound indicates the presence of a mass, while a high-pitched sound indicates hollow areas. To illustrate the ability to image the mechanical properties of single cells, we cultured human mesenchymal stem cells (hMSC, see Methods) on a 300 nm titanium film, supported by a transparent sapphire window (Fig. 1). We used PU to generate and detect picosecond acoustic pulses with optical pump and probe pulses focused on the bottom of the metal film (see Fig. 1 and Methods). Thanks to thermoelastic conversion at the titanium-sapphire interface, the absorption of the femtosecond pump pulses emitted from the first laser launches a broadband acoustic pulse, with a spectrum extending up to 100 GHz. The acoustic pulse propagates through the metal film, and reflects off the titanium-cell interface, thereby reading the mechanical properties of the observed cell. When reaching back the bottom of the titanium film after reflection, the acoustic strain pulse is monitored by the optical-probe pulses emitted from the second laser using elasto-optic coupling (see Methods). This procedure is equivalent to a GHz percussion at the bottom of the metal film to evaluate the mechanical properties of the cell cultured on the opposite side at a nanoscale.

Although the generation mechanism is comparable to that used for MHz photo-acoustic imaging^28^, we implemented it here with much higher acoustic frequencies, and we used the metal film as a photo-acoustic transducer to avoid direct light absorption by the cell. Because of its high-diffusivity, the supporting sapphire layer acts as a heat sink and the titanium film is thick enough to ensure that the cell remains thermally insulated (see Methods)^29^. This key design of the transducer ensures that no laser radiation reaches the top of the titanium film, leaving the cell undisturbed and insulated from laser heating. Contrary to contact-based techniques^7^,^8^, this all-optical approach is contactless and noninvasive.

This set-up works as an inverted pulsed opto-acoustic microscope (iPOM), particularly well-suited to the remote study of cells adhering to the bottom of a culture dish. The diameter of the optical probe spot sets the lateral acoustic resolution, comparable to conventional diffraction-limited optical imaging techniques. With the present laser wavelength, 1030 nm, and objective lens magnification, ×50 (NA 0.8), the diameter of the probe spot is ≈ 2 *µ*m (at 1/e). The lateral resolution can be improved by increasing the numerical aperture or by decreasing the laser wavelength. Since the images are formed by the acoustic reflection coefficient at the cell-metal interface, the technique does not resolve the in-depth structure of the cells. The acoustic reflection coefficient depends on the mechanical properties of the cell probed over distances of the order of the acoustic wavelength, typically 50 to 200 nm in the frequency range we used. Additionally, sensitivity to mechanical resonances of the cell allow mapping cell thicknesses as small as 10 nm, as detailed below. Such thicknesses are 100 times smaller than that accessible by acoustic microscopy in cells^19^. The high resolution capabilities of iPOM allows the study of the structure of single cells and of cell-substrate interactions.

We combined the iPOM with a conventional optical microscope to allow simultaneous top-view white light illumination and fluorescent observation (Fig. 2). For illustration, we selected the nuclear region and the lamellipodium of a typical polarized cell during migration (bottom and top squares in Fig. 2, respectively). The top-view white-light image (Fig. 2a) suggests that the cell height is maximum in the nuclear region and decreases dramatically in the peripheral ruffles. The fluorescence image (Fig. 2b) reveals the presence of actin (green), vinculin (red) and DNA (blue). Bundles of actin filaments, also called stress fibers, appear as bright green stripes perpendicular to the cell edges. One end of each stress fiber is interweaved into the actin cortex near the nucleus^30^. The other end is connected to an adhesion site at the outer edge of the lamellipodium that provides local anchoring on the substrate^31^. The connection of the stress fiber to the substrate forms a contractile structure that is continuously remodeled to allow displacement of the cell in a treadmill-like motion^32^. This complex machinery is key to fundamental cellular processes involving motility or changes in cell shape, such as migration, proliferation or morphogenesis^32^,^33^.

We scanned the sample with iPOM in a two-dimensional raster pattern to produce time-resolved acoustic images of the nuclear region and of the lamellipodium (indicated by squares in Fig. 2) with a 1 *µ*m step. We implemented asynchronous optical sampling by using two mode-locked femtosecond lasers with slightly different repetition frequencies^34^,^35^. We thereby obtained a fast imaging device (a few seconds per pixel) to capture acoustic propagation in the Ti transducer with a 1 ps resolution and a large frequency bandwidth^36^. Online movies 1 and 2 show raw acoustic data acquired with a 1000 images/ns frame rate under the nucleus and the lamellipodium, respectively. We plotted in Fig. 3a and 3b unprocessed snapshots of the movies in the nucleus and in the lamellipodium at 123 and 115 ps, respectively. The very high acoustic contrast reveals the structure of the nucleus, the actin network and the fine details of the complexity of the adhesion sites at the edge of the lamellipodium.

To scrutinize the contribution of these structures, we analyzed the frequency content of the reflected acoustic pulses. We developed a multiscale time-frequency analysis based on a wavelet transform of the transient acoustic signal. We normalized the pulse amplitude to its value in the bare titanium regions to obtain the acoustic reflection coefficient *R_ac_* and to bring out the acoustic contrast (see Methods). We plotted *R_ac_* at 10, 30 and 85 GHz in the nuclear region (Fig. 4, left column) and in the lamellipodium (Fig. 4, right column). When *R_ac_* ~ 1 over the whole bandwidth (reddish areas), all the frequencies are reflected equally. Continuing the analogy with medical percussion, this is equivalent to a frequency-rich, high-pitched sound, revealing hollow regions where bare titanium is probed. Conversely, *R_ac_* < 1 corresponds to a mat sound where high frequencies are attenuated, indicating that the cell is probed.

At 10 GHz, the nuclear region is clearly visible, as is the fibrous structure of the lamellipodium. At 30 GHz, the edges of the cell are well defined and the peripheral ruffles exhibit a rich contrast pattern that reveals the presence of adhesion sites. At 85 GHz, the acoustic contrast within the cell decreases, but the contour of the cell is still clearly observed. The contrast in acoustic images at a given frequency corresponds to spatial variations in cell impedance. For instance, the image of the lamellipodium at 30 GHz shows that in the actin cortex near the nucleus, area A, *R_ac_* ~ 0.65, while in the lamella, area B, *R_ac_* ~ 0.85. This indicates that the actin cortex has a larger acoustic impedance, i.e. it is more rigid (or more dense), than the fibrous lamella, as already observed by acoustic microscopy^14^. However, the frequency dependence of the acoustic contrast indicates that other mechanisms are at work. To understand this frequency dependence, we propose a multicontrast analysis that reveals contributions of reduced cell thickness in the peripheral regions, and of cell-metal bonding.

The comparison of acoustic images at various frequencies reveals dark blue areas in the acoustic images that appear only in very narrow frequency ranges. Indeed when the cell thickness becomes comparable to the acoustic wavelength, the acoustic wave impinging on the Ti-cell interface triggers an acoustic resonance of the cell. In this case, acoustic transmission to the cell is enhanced, causing clear dips in the reflection coefficient *R_ac_* at the resonance frequency. This is illustrated in Fig. 5c. The thinner the cell, the higher the resonance frequency. At 10 GHz, resonances are visible around the nucleus. As the frequency increases, resonances shift toward the thinner edges of the cell, as seen in the 30 GHz acoustic image, revealing areas as thin as 10 nm. These resonances allow the mapping of the mechanical properties of the thinner regions of the cell (see Supplementary Information) not accessible by other techniques.

Weak contact forces between the cell and the supporting substrate can cause frequency dispersion at a nanoscale^37^. The balance between adhesive and repulsive forces forms a well of potential energy per unit area, 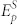, and maintains the cell at an equilibrium position. The acoustic wave impinging on the interface perturbs this equilibrium, and the cell appears as connected to the titanium layer by a massless spring with a stiffness per unit of length, 
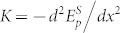
38. As a consequence, the interface induces a frequency dependence of the acoustic reflection *R_ac_* (Fig. 5b), and behaves as an acoustically thin layer of impedance *Z_i_* = *K*/2*πf*. This is particularly visible in the lamellipodium when comparing acoustic images taken at 30 and 85 GHz. In the actin cortex, area A, *R_ac_* increases from 0.65 at 30 GHz to 0.8 at 85 GHz, indicating poor contact between the cell and Ti. On the contrary, in the fibrillar area, area B, *R_ac_* remains constant between 30 and 85 GHz, indicating intimate contact (Fig. 5a). Consideration of this frequency dependence allows mapping of the interfacial stiffness *K* (see Supplementary Information). This stiffness contrast is clearly connected to the nanoscale architecture of the adhesive complexes of the cell.

In conclusion, we have demonstrated a new microscopy method based on the reflection of laser-generated GHz acoustic waves at a cell-metal interface. This technique allows probing the mechanics of adherent cells at a sub-micron scale without contact with the cell. A particular strength of our technique is the implementation of short acoustic pulses that allow probing of the cell in the 10–100 GHz frequency range. The frequency-dependence gives access to the thickness and mechanical properties of the cell, as well as the rigidity of interface at a nanoscale. We have demonstrated the method on migrating hMSCs and have shown that acoustic images can reveal the structure of the nucleus, the fine details of the actin network and of the adhesion pattern, based on elastic properties as the contrast mechanisms.

This ability of iPOM to observe at multiple scales and with multiple contrasts the fibrillar structure of the cell and adhesion sites should allow the assessment of the rigidity of individual actin bundles and the mapping of the adhesion strength at each adhesion site. For this, iPOM fills a significant gap between contact-based techniques that probe the mechanical properties averaged over the whole cell, and imaging techniques that evaluate optical properties at a sub-cell scale. iPOM should therefore prove to be a powerful tool to investigate cell motility, morphogenesis and mechanosensing. In addition, this technique is non-invasive, and shows the potential to perform real-time imaging of the mechanical properties of live cells. The filiation of image formation with medical ultrasonography thus suggests the possibility of transferring diagnostic and imaging applications of ultrasonic imaging to the single-cell scale.

## Methods

### GHz ultrasonography

Conventional PU techniques rely on a mechanical translation stage to control the time delay between synchronous pump and probe pulse trains, and thereby perform time-resolved measurements. This design results in lengthy acquisition times (from tens of minutes to several hours per pixel), and requires a complex manipulation of the laser beams that hinders its use in practical physiological conditions. As an alternative to mechanically-controlled delays, we implemented asynchronous optical sampling by using two mode-locked femtosecond lasers with slightly different repetition frequencies^39^.

**Figure 1:**
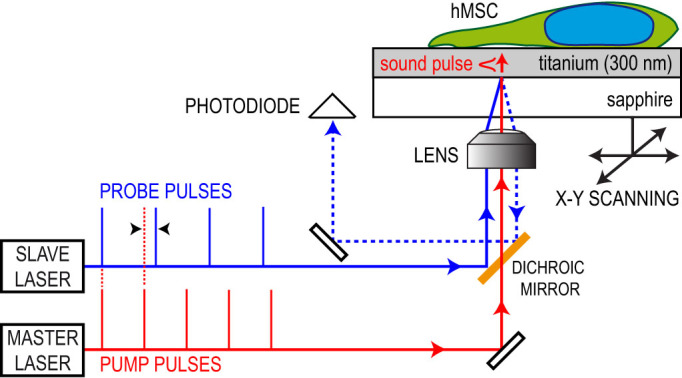
Schematic of the experimental set-up. Side-view of the inverted pulsed opto-acoustic microscope (iPOM). We used two lasers with a slightly different repetition rate to generate broadband acoustic waves and detect echoes in the Ti transducer by optical sampling.

The experimental set-up is described in Fig. 1. We use a compact dual-oscillator (t-Pulse Duo, Amplitudes Systèmes, France) that contains two diode-pumped passively mode-locked Yb:KYW laser cavities. The lasers emit trains of pulses with a duration ~ 400 fs. The master laser, with a repetition frequency of *f_m_* = 50 MHz, is used as a pump. Each pump pulse (wavelength 1040 nm) is absorbed in Ti in the vicinity of the sapphire-Ti interface over a distance comparable to the optical skin depth ~ 15 nm. The ensuing ultrafast thermal dilatation launches a longitudinal coherent-phonon pulse, with a broad spectrum extending up to ~ 100 GHz.

The acoustic strain pulses are detected by the optical probe pulses emitted by the slave laser (wavelength 1028 nm) through acousto-optic coupling. Since the optical skin depth in Ti is smaller than the distance over which the acoustic strain pulse spans, ~ 140 nm (see Supplementary information), the measured optical reflectivity is directly proportional to acoustic strain^40^. The slave laser has a repetition frequency *f_s_* = *f_m_* + Δ*f* that is slightly shifted by Δ*f* = 500 Hz to acquire a time-resolved optical reflectivity variation in 1/Δ*f* = 2 ms. Accurate synchronization of the slave repetition frequency allows exploring a 1/*f_m_* = 20 ns time window with a ≈ 1 ps resolution, that is a spectral bandwidth extending up to 1 THz with a 50 MHz resolution. The high sensitivity of this device allows detecting relative variations in optical reflectivity as small as 10^−5^ with a throughput of 8 pixels/min.

The low-energy coaxial pump (energy 0.2 nJ) and probe (20 pJ) beams are focused through a sapphire plate at the sapphire-Ti interface by a ×50 objective lens to spots of diameters ~ 2 *µ*m (at 1/e). In this configuration, no laser light reaches the Ti-cell interface, making this approach completely non invasive^29^. The lamellipodium and the nucleus of the cell are imaged separately in a raster fashion over a 60 × 60 *µ*m^2^ area. Note that with a smaller laser wavelength such as 400 nm, easily attainable with commercial lasers, and an objective with a higher numerical aperture, the lateral resolution could be as small as 200 nm.

### Design of the opto-acoustic transducer

Each pump pulse is absorbed at the bottom of the metal layer over a depth comparable to the optical skin depth ~ 15 nm^41^. Conduction band electrons are excited and diffuse over a depth of ~ 2 nm during their thermalization with the lattice^29^. The transducer thickness is designed so that these overheated electrons do not reach the top Ti surface where cells are cultured. The heat then diffuses on a larger scale into the bulk of the Ti film and of the sapphire substrate on a nanosecond timescale. The sapphire supporting substrate was chosen for its optical transparency at the laser wavelength, for its low acousto-optic coupling, preventing elastic waves propagating in sapphire from perturbing the optical reflectivity, and for its high thermal diffusivity acting as a heat sink. Thermal waves thus do not reach the Ti top surface, keeping the cell thermally insulated from laser heating.

### Wavelet analysis

To investigate the amplitude variation of the acoustic echoes across the cell as a function of the frequency, we perform a time-frequency analysis. The wavelet transform is the perfect tool to analyze a time pulse with a varying shape. In our configuration indeed, the shape of the echo depends on the acoustic reflection at an heterogeneous interface with a low interfacial stiffness. We convolve the measured change of optical reflectivity *δR*(*t*) with a Morlet wavelet^29^. We define the amplitude of the reflected acoustic pulse at the center frequency of the wavelet as the maximum of the energy distribution. This simple procedure yields the amplitude of the reflected acoustic pulse at frequencies ranging from 10 to 85 GHz. Below this range the first pulse starts to overlap with the second pulse that has reflected twice at the Ti-cell interface, given the thickness ~ 300 nm of the Ti film. Beyond 85 GHz, the signal-to-noise ratio prevents clear identification of the pulse. In order to solely observe the contribution of the cell to the frequency dependence, we normalize the energy spectrum obtained at each position by the energy spectrum of the acoustic pulse reflected in the bare Ti region (averaged over several points). This normalization yields the acoustic reflection coefficient *R_ac_*(*f*).

### Cell culture and reagents

Primary human (bone marrow) mesenchymal stem cells (Lonza, Switzerland) were cultured in minimum essential medium (Alpha-MEM, Gibco) supplemented with 10% (vol/vol) FBS, 1% penicillin/streptomycin and incubated in a humidified atmosphere containing 5% (vol/vol) CO_2_ at 37°C. All cells were used at low passage numbers (passage 4 to 8). Cells were subconfluently cultured and plated at 10^4^ cells/cm^2^. After a 24 hour culture, the cells on the surfaces were fixed for 30 min in 4% paraformaldehyde/PBS at 4°C. After fixation, the cells were permeabilized in 1% Triton X-100 in PBS for 15 min. Actin and vinculin were visualized by treating the cells for 1 hour at 37°C with 1% (vol/vol) phalloidin-FITC (Sigma) and with mouse monoclonal anti-vinculin (Invitrogen), respectively. The cells were then incubated with an Alexa Fluor 588-conjugated F(ab)2 fragment of rabbit anti-mouse IgG(H+L) for 30 minutes at room temperature. The cell nuclei were counterstained in 20 ng/mL DAPI for 10 min at room temperature. The images were produced using a Leica DM5500B epifluorescence microscope and MetaMorph software. Images were taken at ×40 magnification.

## Author Contributions

T.D. and B.A. conceived and designed the experiments. M.A.G. performed the experiments. T.D. and M.A.G. analyzed the data. T.D. wrote the paper. O.F.Z. cultured the cells and performed the fluorescent imaging. J.M.R., Y.G. and S.D. designed and implemented the asynchronous optical sampling device. M.C.D. and B.A. conceived and supervised the project. All authors commented on the manuscript.

## Supplementary Material

Supplementary Information Supplementary info

Supplementary Information Movie 1

Supplementary Information Movie 2

## Figures and Tables

**Figure 2:**
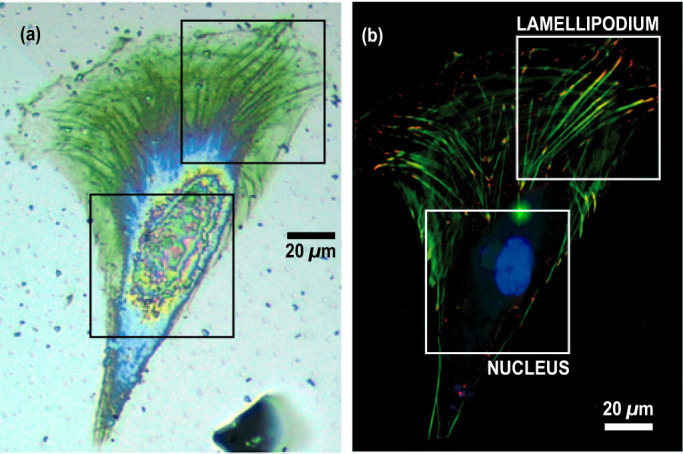
hMSC optical images. (a), Top-view white-light image. (b), Fluorescent image showing actin (green), vinculin (red) and nucleus (blue). Areas scanned with iPOM are indicated by squares. Scale bar: 20 *µ*m.

**Figure 3:**
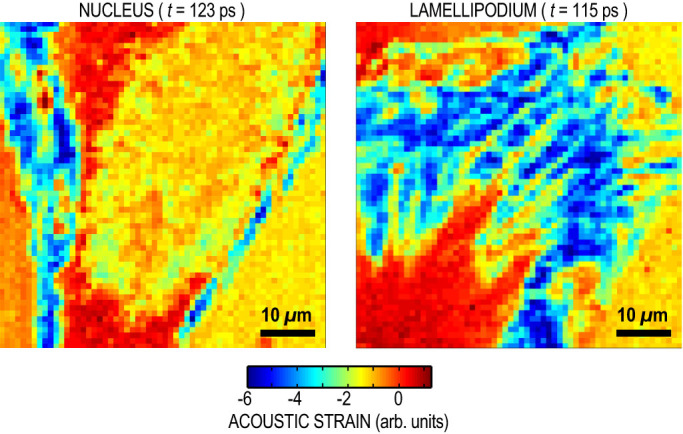
Unprocessed acoustic images. Raw acoustic images of the nuclear region (left) and lamellipodium (right) taken at times 123 and 115 ps, respectively. Scale bar: 10 *µ*m.

**Figure 4:**
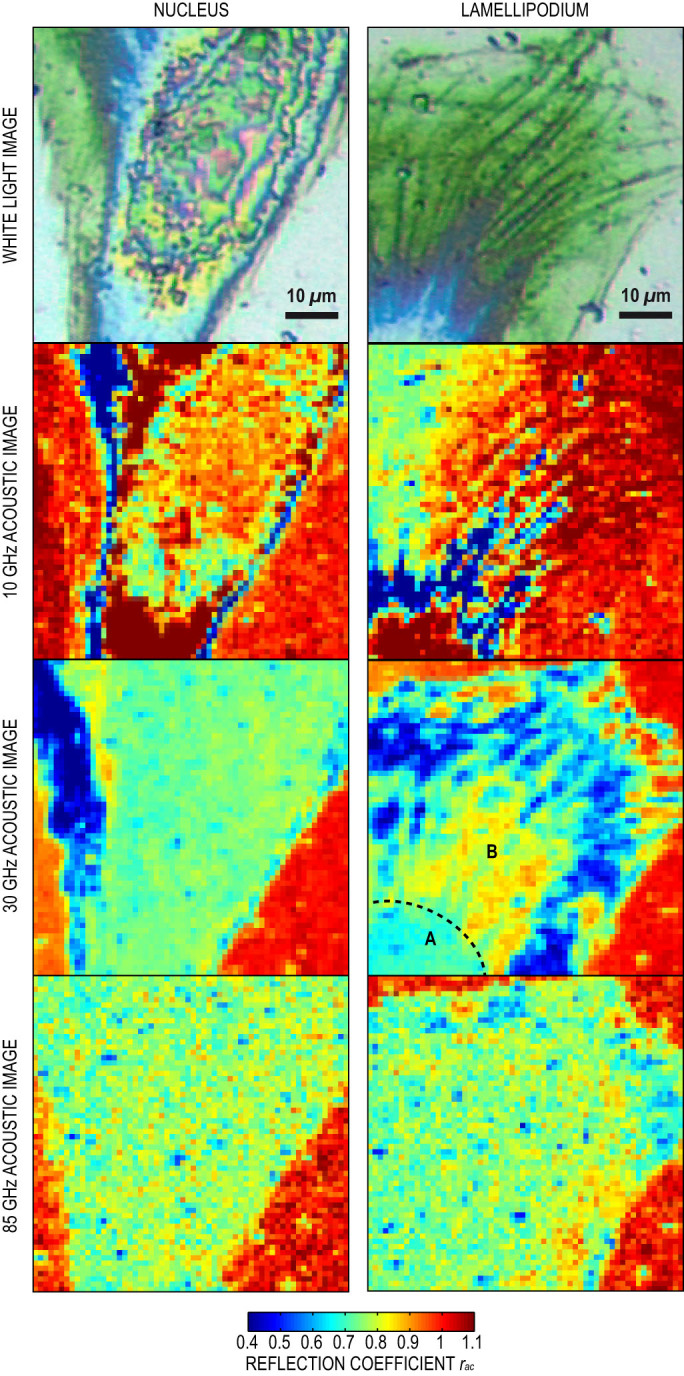
Acoustic images. Images of the nuclear region (left column) and lamellipodium (right column). The first row shows white-light images for comparison. The subsequent rows show acoustic images taken at 10, 30 and 85 GHz, respectively. The scale is identical on all images (scale bar of 10 *µ*m). The very high acoustic contrast allows the contour of the cell, the structure of the nucleus and the fine details of the fibrillar lamellipodium to be clearly observed. Variation of the acoustic reflection coefficient *R_ac_* with frequency allows analysis of the contacts at a nanoscale. For instance, areas A and B labelled in the 30 GHz image indicate areas of poor and good contact, respectively (see text).

**Figure 5:**
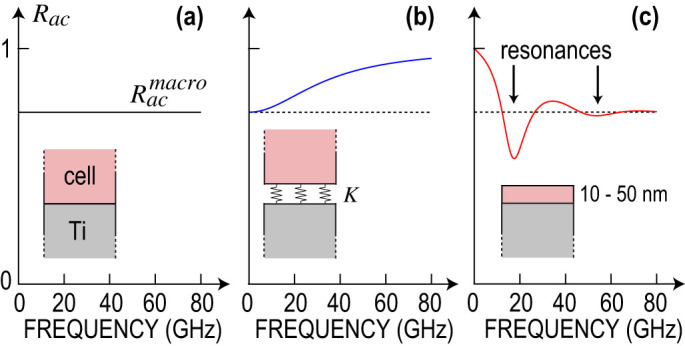
Frequency dependence of the acoustic reflection coefficient. Acoustic reflection coefficient *R_ac_* for typical titanium-cell interfaces. (a), Perfect contact of a thick cell: 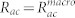 does not depend on *f*. (b), Imperfect contact of a thick cell: *R_ac_* increases from 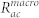 to 1 with increasing *f*. (c), Perfect contact of a thin cell: *R_ac_* shows dips at resonant frequencies of the cell, and tends towards 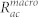 at high frequencies.
