# Supplementary material for: All-optical broadband ultrasonography of single cells
Source: Sci Rep. 2015 Mar 3;5:8650. doi: 10.1038/srep08650 (PMC4346798; doi:10.1038/srep08650)
Supplement: Supplementary Information — Supplementary info [file srep08650-s1.pdf]

Supplementary information for the paper "All-optical broadband ultrasonography of single cells"

T. Dehoux,[1,2] M. Abi Ghanem,[1,2] O. F. Zouani,[3] J.-M. Rampnoux,[4]
Y. Guillet,[1,2] S. Dilhaire,[4] M.-C. Durrieu,[3] B. Audoin[1,2]

[1] *Univ. Bordeaux, I2M, UMR 5295, F-33400 Talence, France*

[2] *CNRS, I2M, UMR 5295, F-33400 Talence, France*

[3] *Univ. Bordeaux, CBMN, UMR CNRS 5248, F-33607 Pessac, France and*

[4] *Univ. Bordeaux, LOMA, CNRS UMR 5798, F-33400 Talence, France*

TRANSIENT REFLECTIVITY

Figure S1a shows the optical reflectivity change δR in the bare Ti region (black plain line), and at the center of the cell nucleus (red dotted line). At time $t = 0$ we observe a sudden rise (~ 1 ps) in δR due to overheated electrons generated by the laser absorption in Ti in the vicinity of the Ti-Al_2O_3 interface. This is followed for $t > 0$ by a slower decrease with a characteristic time of 0.1 ns because of heat diffusion, involving frequencies greater than the laser repetition frequency $f_m = 50$ MHz. These waves diffuse in Ti over a length $\lesssim (D/2\pi f_r)^{1/2} \approx 70$ nm, where $D = 1.4 \times 10^{-6}$ m^2/s is the diffusivity of Ti [1]. Owing to its high diffusivity, the Al_2O_3 supporting layer acts as a heat sink and reduces further heat diffusion in the Ti film. The contributions to the reflectivity arising from overheated electrons and from thermal diffusion remain unaffected by the presence of the cell in the probed area. This demonstrates that the thermal waves of frequencies greater than 50 MHz, as well as overheated electrons, do not reach the Ti top surface. The thickness of the Ti layer is thus well designed, and the cell is thermally insulated from laser heating.

In addition to these thermal contributions, a compressive stress field is generated at $t = 0$ in the Ti film over the optical skin depth ≈15 nm.[2] Owing to the stress generation at an interface between two solids, a unipolar compressive acoustic pulse is launched perpendicularly to the Ti-Al_2O_3 interface in each material.[3] In the Ti film the acoustic pulse is reflected from the Ti free surface (or Ti-cell interface) with a reflection coefficient R_{ac}. It is detected at the Ti-Al_2O_3 interface as an echo at time ∼85 ps. Successive reflections at the film boundaries give rise to a second echo detected at time ∼170 ps. The echoes are

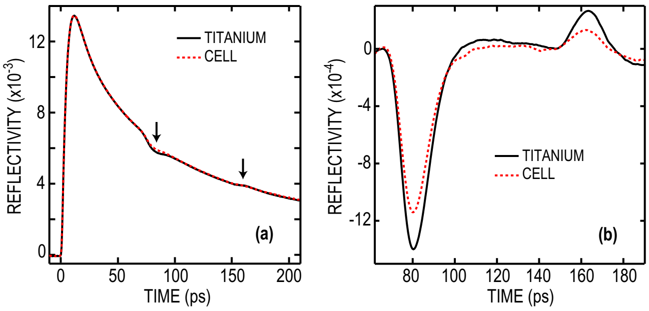

FIG. S1. **Transient reflectivity. a,** Optical reflectivity change probed in the bare Ti area (black plain line) and in the cell area (red dashed line). Acoustic echoes are indicated with arrows. **b,** Zoom-in on the acoustic echoes, with a polynomial subtracted for clarity.

indicated with arrows in **Fig. S1a**. To remove the contribution of thermal relaxation in Ti and to observe solely the acoustic pulses, we subtract a polynomial. A zoom-in on the acoustic pulses is plotted in **Fig. S1b**. We then plot the result over the scanned area, and play these images as a function of time to produce supplementary movies (see online).

We detect the acoustic strain pulses, $\eta(z,t)$, through acousto-optic coupling. We measure variations in the optical reflectivity, which are proportional to the integral of the acoustic strain over the probe skin depth[4]. As observed in **Fig. S1b**, the time-envelop of the strain pulse can be approximated by a Gauss function of full width at half maximum $\tau \approx 20$ ps. The strain pulse thus spans over a distance $\xi = \tau V \sim 140$ nm, where $V = 7$ nm/ps is the sound velocity in Ti[5]. Since ξ is greater than the probe skin depth ~ 15 nm, the optical reflectivity is directly proportional to the amplitude of the strain pulse[4]. The images of the optical reflectivity we have shown in the manuscript can thus be interpreted as acoustic strain images.

CELL IMPEDANCE AND INTERFACIAL STIFFNESS

Consider the cell as an homogeneous medium in the axial direction. The propagation of acoustic waves is mediated by the local oscillation of the stress σ at frequency f. This stress sets the particles in motion at a velocity v around their equilibrium position. Similarly to a transfer function, the acoustic impedance, Z, relates the imposed stress to the induced velocity, $Z = \sigma/v$. At a macroscale, the reflection of acoustic waves at an interface between the titanium film and an acoustically thick cell (few microns) is dominated by the mismatch of acoustic impedances, and does not depend on f (**Fig. S2a**). If the impedance of the cell matches that of Ti, there is no acoustic reflection and $R_{ac} = 0$. Conversely, if the cell is much softer than Ti, the reflection is total and $R_{ac} = 1$. On the face of it, acoustic images at a given frequency could be understood in this frame. However, the acoustic contrast depending on frequency indicates that other mechanisms, inherent to ultrasonography at a nanoscale, need to be accounted for.

When the thickness of the cell, d, becomes comparable to the acoustic wavelength, λ, the acoustic wave impinging on the Ti-cell interface triggers an acoustic resonance of the cell. In this case, acoustic transmission to the cell is enhanced, causing dips in the reflection coefficient R_{ac} at the resonance frequency f_R (higher harmonics are strongly attenuated

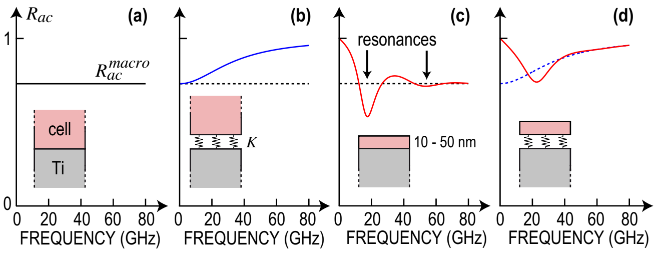

FIG. S2. **Frequency dependence of the acoustic reflection coefficient.** Acoustic reflection coefficient R_{ac} [6] for typical titanium-cell interfaces. **a,** Perfect contact of a thick cell: $R_{ac} = R_{ac}^{macro}$ does not depend on f. **b,** Imperfect contact of a thick cell: R_{ac} increases from R_{ac}^{macro} to 1 with increasing f. **c,** Perfect contact of a thin cell: R_{ac} shows dips at resonant frequencies of the cell, and tends towards R_{ac}^{macro} at high frequencies. **d,** Imperfect contact of a thin cell: R_{ac} shows dips at resonant frequencies of the cell, and tends towards R_{ac} for an imperfect contact at high frequencies.

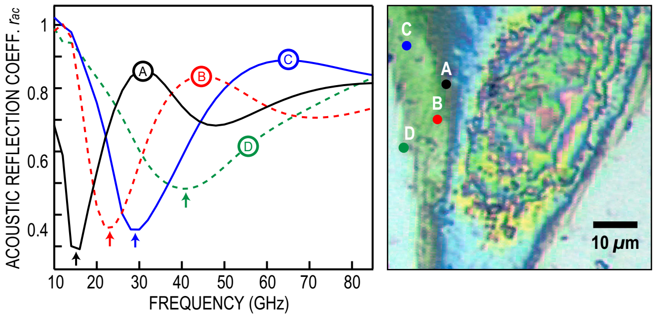

FIG. S3. **Mechanical resonances.** Acoustic reflection coefficient R_{ac} vs frequency (left) measured in thin regions at the edge of the cell. The resonance frequencies f_R are indicated by upward arrows. The corresponding location of the measurement points are indicated on the optical image (right).

due to cell viscosity) that appear as dark blue areas in the acoustic images (**Fig. 4** of the manuscript). In addition, imperfect cell-metal contact can induce frequency dispersion that overlap with mechanical resonances. For this, it is difficult to disentangle the two phenomena. In the following, we propose a preliminary analysis that allows investigating mechanical resonances and interfacial defects in separate regions of the cell by considering distinct frequency ranges.

To illustrate mechanical resonances, we plot in **Fig. S3** the reflection coefficient R_{ac} as function of the acoustic frequency (left) in thin regions at the edge of the cell (indicated by

letters A to D on the righthand-side image). We observe clear dips in R_{ac} at the resonant frequency f_R (indicated by upward arrows). As the cell becomes thinner towards its outer edges, f_R increases. At very low frequencies, $R_{ac} \approx 1$ since the cell is acoustically transparent, $\lambda \gg d$. In areas where we observe high-amplitude resonances, we plot the resonance frequency f_R in **Fig. S4**, overlaid with the white-light image.

The resonance frequency f_R depends on the interfacial stiffness K and on the thickness of the cell. Assuming that the cell and the Ti film are tightly bonded, $K \to \infty$, the cell behaves as a quarter-wave resonator (since the acoustic impedance of Ti is much larger than that of the cell) and resonates at odd harmonics of the fundamental frequency $f_R = V_c/4d$, where V_c is the sound velocity in the cell. For imperfect nanoscale interfaces, the fundamental frequency increases toward $f_R = V_c/2d$ as the cell behaves asymptotically as a half-wave resonator (**Fig. S2d**).

For illustration, we plot in **Fig. S4** the thickness estimated from the quarter-wave resonator, $f_R = V_c/4d$, considering a typical sound velocity in the cell, $V_c = 3700$ m/s.[5] This reveals the thinner regions of the cell, mostly located in the lamellipodium. The pattern of nano-thickness regions matches the white-light image, demonstrating that the technique is indeed sensitive to the nano-thickness of cells. In areas where resonance frequency is high, $f_R > 15$ GHz, the cell is thin, $d \lesssim 60$ nm, and there are no actin-rich components, as confirmed by the fluorescent image. In regions where the cell is thicker, resonances are on the low-frequency range of the acoustic bandwidth ($f_R < 15$ GHz).

To analyze the frequency-dependence of R_{ac} due to the imperfect cell-metal contact, we consider a simple model describing a titanium half-space, of calibrated impedance $Z_t =$ 32 MPa.s/m, connected to a thick cell of impedance Z_c by a massless spring of stiffness per unit length K (**Fig. S2b**).[5] Using this formalism, the acoustic reflection coefficient is then:[7]

$$R_{ac} = \left| \frac{Z_t - Z_c + i\omega Z_t Z_c/K}{Z_t + Z_c + i\omega Z_t Z_c/K} \right| \tag{1}$$

where Z_c and ω are the acoustic impedance of the cell and the angular frequency, respectively. This formula shows that the variation of R_{ac} across the cell can be attributed to changes in the cell impedance, owing to rigidity and density fluctuations within the cell. It also demonstrates that a weak Ti-cell contact would result in an increase in R_{ac} with increasing frequency, from the reflection coefficient for a perfect interface $R_{ac}^{macro} = (Z_t - Z_c)/(Z_t + Z_c)$ at low frequencies to that of a free surface $R_{ac} = 1$ at high frequencies, as shown in **Fig. S2b**.

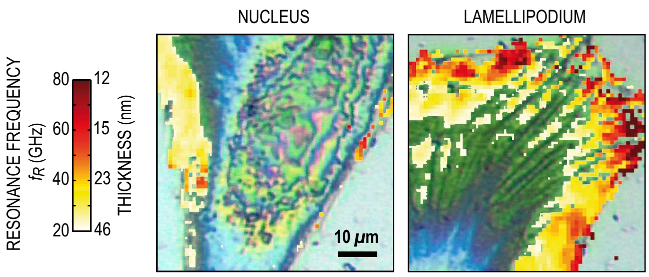

FIG. S4. **Acoustic images.** White-light images of the cell overlaid with the cell thickness measured from the mechanical resonance frequency.

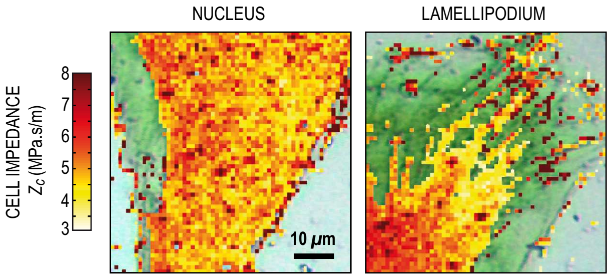

FIG. S5. **Acoustic images.** White-light images of the cell overlaid with the cell impedance obtained by fitting the frequency dispersion of R_{ac}.

Equation 1 can easily be modified to include the finite thickness of the cell (**Fig. S2d**). In this case we see that far enough from the resonances, R_{ac} asymptotically tends to the behavior predicted for an acoustically thick cell (blue dashed line in **Fig. S2d**).

Thus in thick regions of the cell, $f_R < 15$ GHz, the high-frequency dependence from 40 to 80 GHz of the acoustic reflection R_{ac} is mostly due to interfacial imperfections (**Fig. S2d**). We therefore fit R_{ac} with Eq. 1, and we plot Z_c and K that give the best fit to the data in **Figs. S5** and **S6**, respectively, overlaid with the white-light image. For tightly bonded interfaces, a large stress is required to induce a small separation distance and $K \rightarrow +\infty$, as observed along the fiber in the lamellipodium (**Fig. S6**). In this area where K reaches 15 GPa/nm, the adhesion of the cell with the metal is the strongest to provide anchor for the tread-milling motion during migration [8]. At the same time, in this area the impedance is the lowest, whereas the impedance increases gradually to 5 MPa.s/m in the nucleus due to molecular crowding [5].

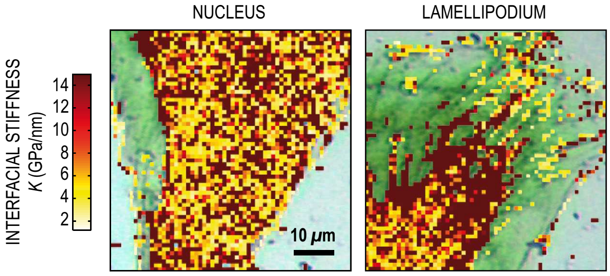

FIG. S6. **Acoustic images.** White-light images of the cell overlaid with interfacial stiffness obtained by fitting the frequency dispersion of R_{ac}.

[1] Dehoux, T. & Audoin, B. Non-invasive optoacoustic probing of the density and stiffness of single biological cells. *J. Appl. Phys.* **112**, 124702 (2012).

[2] Audoin, B. *et al.* Picosecond acoustics in vegetal cells: Non-invasive in vitro measurements at a sub-cell scale. *Ultrasonics* **50**, 202–207 (2010).

[3] Dehoux, T., Wright, O. B., Voti, R. L. & Gusev, V. E. Nanoscale mechanical contacts probed with ultrashort acoustic and thermal waves. *Phys. Rev. B* **80**, 235409 (2009).

[4] Gusev, V. E. & Karabutov, A. A. *Laser optoacoustics* (American Institute of Physics, New York, 1993).

[5] Abi Ghanem, M. *et al.* Remote opto-acoustic probing of single-cell adhesion on metallic surfaces. *J. Biophotonics* (2013).

[6] Auld, B. *Acoustic fields and waves in solids*, vol. 1 (R. E. Krierger Publishing Compagny, Malabar, Florida, 1990).

[7] Tattersall, H. G. The ultrasonic pulse-echo technique as applied to adhesion testing. *J. Phys. D* **6**, 819–832 (1973).

[8] Pantaloni, D., Clainche, C. L. & Carlier, M.-F. Mechanism of actin-based motility. *Science* **292**, 1502–1506 (2001).
